# Advancing precision agriculture with deep learning enhanced SIS-YOLOv8 for Solanaceae crop monitoring

**DOI:** 10.3389/fpls.2024.1485903

**Published:** 2025-01-09

**Authors:** Ruiqian Qin, Yiming Wang, Xiaoyan Liu, Helong Yu

**Affiliations:** ^1^ College of Information Technology, Jilin Agricultural University, Changchun, China; ^2^ Teaching Resource Information Service Center, Changchun Institute of Education, Changchun, China; ^3^ Changchun Sci-Tech University, Changchun, China

**Keywords:** deep learning, detection of diseases, object detection, YOLOv8, digital agriculture

## Abstract

**Introduction:**

Potatoes and tomatoes are important Solanaceae crops that require effective disease monitoring for optimal agricultural production. Traditional disease monitoring methods rely on manual visual inspection, which is inefficient and prone to subjective bias. The application of deep learning in image recognition has led to object detection models such as YOLO (You Only Look Once), which have shown high efficiency in disease identification. However, complex climatic conditions in real agricultural environments challenge model robustness, and current mainstream models struggle with accurate recognition of the same diseases across different plant species.

**Methods:**

This paper proposes the SIS-YOLOv8 model, which enhances adaptability to complex agricultural climates by improving the YOLOv8 network structure. The research introduces three key modules: 1) a Fusion-Inception Conv module to improve feature extraction against complex backgrounds like rain and haze; 2) a C2f-SIS module incorporating Style Randomization to enhance generalization ability for different crop diseases and extract more detailed disease features; and 3) an SPPF-IS module to boost model robustness through feature fusion. To reduce the model’s parameter size, this study employs the Dep Graph pruning method, significantly decreasing parameter volume by 19.9% and computational load while maintaining accuracy.

**Results:**

Experimental results show that the SIS-YOLOv8 model outperforms the original YOLOv8n model in disease detection tasks for potatoes and tomatoes, with improvements of 8.2% in accuracy, 4% in recall rate, 5.9% in mAP50, and 6.3% in mAP50-95.

**Discussion:**

Through these network structure optimizations, the SIS-YOLOv8 model demonstrates enhanced adaptability to complex agricultural environments, offering an effective solution for automatic crop disease detection. By improving model efficiency and robustness, our approach not only advances agricultural disease monitoring but also contributes to the broader adoption of AI-driven solutions for sustainable crop management in diverse climates.

## 1 Introduction

Potatoes and tomatoes are key solanaceous crops in global agricultural production. Their widespread cultivation, significant economic value, and notable nutritional benefits make them crucial to contemporary agricultural practices. In addition, these crops play an integral role in global food security, providing essential calories and nutrients to millions of people worldwide. With the global population steadily increasing, ensuring a stable and sustainable supply of these crops is vital for food security, especially in regions that are highly dependent on potato and tomato cultivation. However, crop diseases like early blight and late blight, which affect potato and tomato foliage respectively, substantially hinder the growth and productivity of these crops, resulting in considerable economic losses (Yuen, 2021; Maurya et al., 2022; Wu et al., 2022; Kang et al., 2023a). Beyond economic concerns, the spread of these diseases also poses risks to food availability, threatening the livelihoods of farmers and exacerbating food insecurity.

Both early and late blight in potatoes and tomatoes are fungal diseases (Brouwer et al., 2023; Gonzalez-Jimenez et al., 2023; Park et al., 2023; Sajeevan et al., 2023; Hyder et al., 2024; Zhang et al., 2024). The pathogen responsible for early blight is Alternaria solani, from the subphylum Pezizomycotina, while Phytophthora infestans, belonging to the subphylum Oomycota, causes late blight. Factors influencing the prevalence of these diseases include climatic conditions, soil type, planting density, and varietal resistance. These diseases are more severe in moist and rainy climates. Moreover, continuous cropping and soil-borne pathogens significantly contribute to the incidence of these diseases (Ricky and Nazari, 2021; Aldakheel et al., 2024; Ames et al., 2024; Saffer et al., 2024; Sarah et al., 2024; Wang and Liu, 2024). Therefore, developing a method for the accurate and timely identification of common diseases in potatoes and tomatoes is crucial for precision agricultural management and enhancing crop yields. Timely detection allows for early intervention, reducing the spread of diseases, minimizing the use of chemical treatments, and ultimately ensuring better crop health and more sustainable farming practices.

Traditional disease monitoring methods have predominantly relied on manual visual recognition. This approach is not only time-consuming and labor-intensive but also subject to variability and subjective judgment. With the rapid advancement of artificial intelligence technology, particularly the application of deep learning in image recognition, a new, efficient, and accurate method for disease monitoring has emerged. Deep learning models, capable of processing extensive arrays of image data, can automatically identify and detect crop diseases, thus significantly enhancing the efficiency and accuracy of diagnosis.

Especially noteworthy among deep learning-based object detection models are the YOLO (You Only Look Once) series algorithms, which have demonstrated immense potential for real-time image processing due to their speed and efficiency. Various researchers have adopted these object detection models; for instance, Eman Abdullah Aldakheel et al. (2024) utilized the PlantVillage dataset—comprising photos of both healthy and diseased plant leaves from 14 different species—to develop a YOLOv4-based system for predicting agricultural diseases. Meanwhile, Zhao et al. (2023) enhanced the YOLOv5s model and introduced a structure known as Channel Attention Module(CAM), which extracts both global and local features from each network layer, thereby improving the model’s ability to detect crop diseases. Yang et al. (2024) employed the upgraded YOLOv7 model to detect minor disease spots on grape leaves. This model incorporates a new detection head for identifying small targets, uses asymmetric convolution for extracting multi-scale features, and includes an improved channel attention mechanism. Additionally, Jia et al. (2023) developed a rice pest and disease identification model using the enhanced YOLOv7 algorithm. This model leverages the lightweight MobileNetV3 network for feature extraction, reduces parameterization, and combines Coordinate Attention (CA) with the Scaled Intersection over Union Loss Function(SIoU) to enhance accuracy.

While the improved deep neural network models discussed previously have demonstrated outstanding performance in target detection tasks for crop diseases, they continue to face numerous challenges when addressing diverse practical issues in agriculture. In real-world agricultural environments, such as during rainy seasons or in hazy conditions, crop leaves often appear against highly disruptive backgrounds. Images captured under these conditions typically contain significant noise (Kang et al., 2023b; Rowe et al., 2024), which leads to a noticeable decline in the robustness and generalization capabilities of deep learning models in practical applications. In terms of data acquisition and labeling, the diversity of plant species means that not all plant data is readily accessible; this is particularly true for data on crop diseases that are difficult to obtain or that require substantial human and material resources to collect. Moreover, data labeling itself demands considerable resources. Therefore, the introduction of domain generalization techniques is crucial for addressing these issues. Domain generalization, a type of transfer learning, allows a model to learn from a specific domain during the training phase and then apply this knowledge to other domains during the testing phase. The introduction of this technique is significant for resolving data acquisition and labeling issues.

Through domain generalization (Wang et al., 2022a), models can learn effective feature representations on limited training data, which can then be applied to other domains during the testing phase. This capability is essential for tasks like disease detection in crops, where models need to generalize across different plant species, growing conditions, and environmental variables. As a result, domain generalization can improve the model’s ability to recognize and diagnose diseases accurately and reliably, even in the presence of domain shifts, thus enhancing the robustness and scalability of disease prediction systems.

Furthermore, current mainstream models for detecting crop diseases often focus on improving the detection accuracy of diseases in a single crop, neglecting the model’s capability to recognize the same disease across different crops. This bias leads to models that perform well in detecting diseases in single crops but often fail to exhibit the necessary robustness and generalization capabilities in cross-crop and complex agricultural scenarios. This makes it extremely difficult for models to detect diseases in crops where data is scarce. Therefore, this study proposes the necessity of optimizing existing deep learning architectures to enhance their robustness and generalization capabilities in complex agricultural contexts and across different domains.

To address the challenges outlined previously, this study focuses on the detection of the same diseases in potatoes and tomatoes, introducing the SIS-YOLOv8 model for identifying early and late blight in the leaves of these plants within complex backgrounds. Initially, we selected samples of early and late blight, as well as healthy leaf images from potatoes and tomatoes, using the open-source public dataset PlantVillage. We also gathered a large number of related images through web scraping. These images were annotated to create a comprehensive dataset of early and late blight in potato and tomato leaves. To mimic noise interference typical in agricultural settings, we introduced two types of simulated noise processing, indicative of rain and haze conditions, into the dataset images. Furthermore, we applied image enhancements, including stretching, scaling, and adjustments in the HSV (Hue, Saturation, Value) color space, to bolster the model’s robustness and generalization capabilities.

In response to these challenges, we developed the SIS-YOLOv8 model by introducing key architectural enhancements aimed at improving robustness and generalization across various agricultural scenarios. These enhancements—Fusion-InceptionConv, C2f-SIS, and SPPF-IS—were designed to strengthen the model’s ability to adapt to diverse and complex agricultural environments, such as disease detection in crops. The integration of Style Randomization (Wang et al., 2022b) and Cross-Norm (Tang et al., 2021) further helps the model generalize effectively to different agricultural settings, making it more applicable to real-world farming challenges.

Following these improvements, the model achieved strong performance metrics, with significant gains in precision and recall for agricultural tasks such as disease detection in potatoes and tomatoes. This demonstrates the model’s potential to enhance crop management practices, offering reliable and accurate insights for early detection of plant diseases, which can ultimately reduce losses and improve productivity.

Moreover, through model pruning, we made the SIS-YOLOv8 more efficient and lightweight, making it suitable for deployment in resource-constrained environments, such as on-farm devices or low-power agricultural sensors. Despite the reduction in model size, performance remained high, further underscoring the model’s practicality in real-world agricultural applications.

When compared to other state-of-the-art models like YOLOv9 (Wang et al., 2024b) and YOLOv10 (Wang et al., 2024a), the SIS-YOLOv8 outperforms them in domain generalization, making it a particularly effective tool for diverse agricultural applications. This demonstrates the model’s capacity to adapt to different crops and environmental conditions, providing farmers with a versatile and scalable solution for precision agriculture. Moreover, in a comparative analysis with the two-stage detection model FasterRCNN (Ren et al., 2016), the SIS-YOLOv8 not only shows superior performance in terms of detection accuracy but also excels in processing speed. The FasterRCNN, while effective for certain tasks, tends to be slower due to its two-stage detection pipeline, which involves region proposal generation followed by object classification. In contrast, the SIS-YOLOv8 achieves significantly faster inference times due to its single-stage architecture, which directly predicts bounding boxes and class labels in one step. Our experiments indicate that the SIS-YOLOv8 surpasses FasterRCNN both in speed and efficiency, with a much higher Frames Per Second (FPS) value, demonstrating its suitability for real-time applications in agricultural settings where both accuracy and speed are critical.

This research contributes to the agricultural disease detection field by providing a dataset of common diseases in potatoes and tomatoes, totaling 4,800 images. Additionally, this study introduces a new research approach for disease detection, namely using domain generalization methods from transfer learning to improve the model. We propose an improved SIS-YOLOv8 model, which, through optimizing the network structure, enhances adaptability to complex agricultural environments, resulting in higher accuracy and recall rates in agricultural disease detection tasks. This study employs the DepGraph pruning method (Fang et al., 2023), which trims the model to compress unnecessary parameters, thus making it more lightweight. The pruned model’s mAP50 is 87.5%, mAP50-90 is 84.0%, the accuracy rate is 85.7%, and the recall rate is 82.2%. The model’s parameter count is reduced to 2.41M, with a Frames Per Second(FPS) of value of 295.5, representing a 330% increase in processing speed. The accuracy has increased by 1.6% compared to the pre-pruned model. While reducing the model’s parameter and computational requirements, the mAP has improved by 0.1% compared to the pre-pruned model and by 5.9% compared to the original YOLOv8n model.

## 2 Materials and methods

### 2.1 Materials

#### 2.1.1 Images acquisition

**Figure 1:**
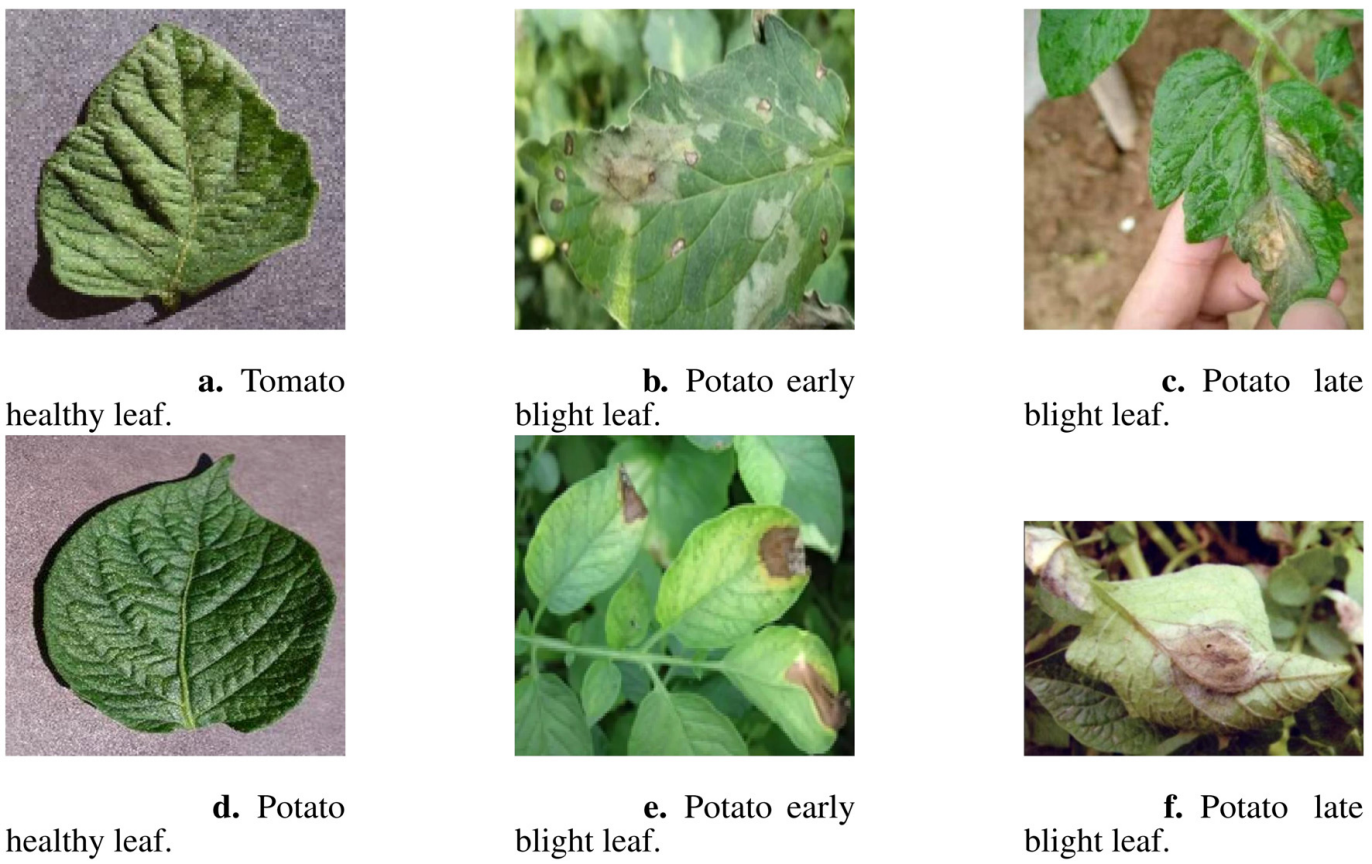
A subset of dataset images showcasing different plant leaf types. **(A)** Tomato healthy leaf, **(B)** Potato early blight leaf, **(C)** Potato late blight leaf, **(D)** Potato healthy leaf, **(E)** Potato early blight leaf, and **(F)** Potato late blight leaf.

Images of potato and tomato leaves exhibiting early blight, late blight, and healthy states were obtained from the publicly available PlantVillage dataset, which includes images captured under controlled laboratory conditions. The PlantVillage dataset is open-source and can be accessed at https://github.com/spMohanty/PlantVillage-Dataset. Additionally, images depicting similar conditions were collected from field environments through web crawling techniques, which involved gathering publicly accessible images from agricultural research websites, plant disease repositories, and online plant health databases. These images were then combined into a comprehensive dataset designed specifically for the detection of early blight, late blight, and healthy conditions in potato and tomato leaves. The dataset consists of 3268 images in total, with 1628 images representing potato leaves and 1640 images representing tomato leaves. A subset of these images is illustrated in **Figure 1**.

The annotation process was conducted using LabelImg software under the guidance of a plant pathology expert. A single annotator, experienced in plant disease diagnosis, was responsible for labeling the images based on the visible symptoms of early blight, late blight, and healthy conditions. It is important to note that some images included a mixture of conditions, such as both healthy and early blight-affected leaves within the same image. During the annotation phase, images were labeled according to the predominant condition visible on the leaf, with each image receiving a label corresponding to early blight, late blight, or healthy.

Inter-annotator reliability was not directly assessed, as only one annotator was involved. However, the annotations were verified by a plant pathology expert to ensure accuracy and consistency across the dataset.

The dataset is split into a training set and a validation set. The training set contains 524 images labeled with early blight, 537 images labeled with healthy, and 567 images labeled with late blight. The validation set includes 554 images labeled with early blight, 534 images labeled with healthy, and 552 images labeled with late blight. This balanced distribution ensures that each class is well-represented for model training and evaluation.

#### 2.1.2 Data augmentation

**Figure 2:**
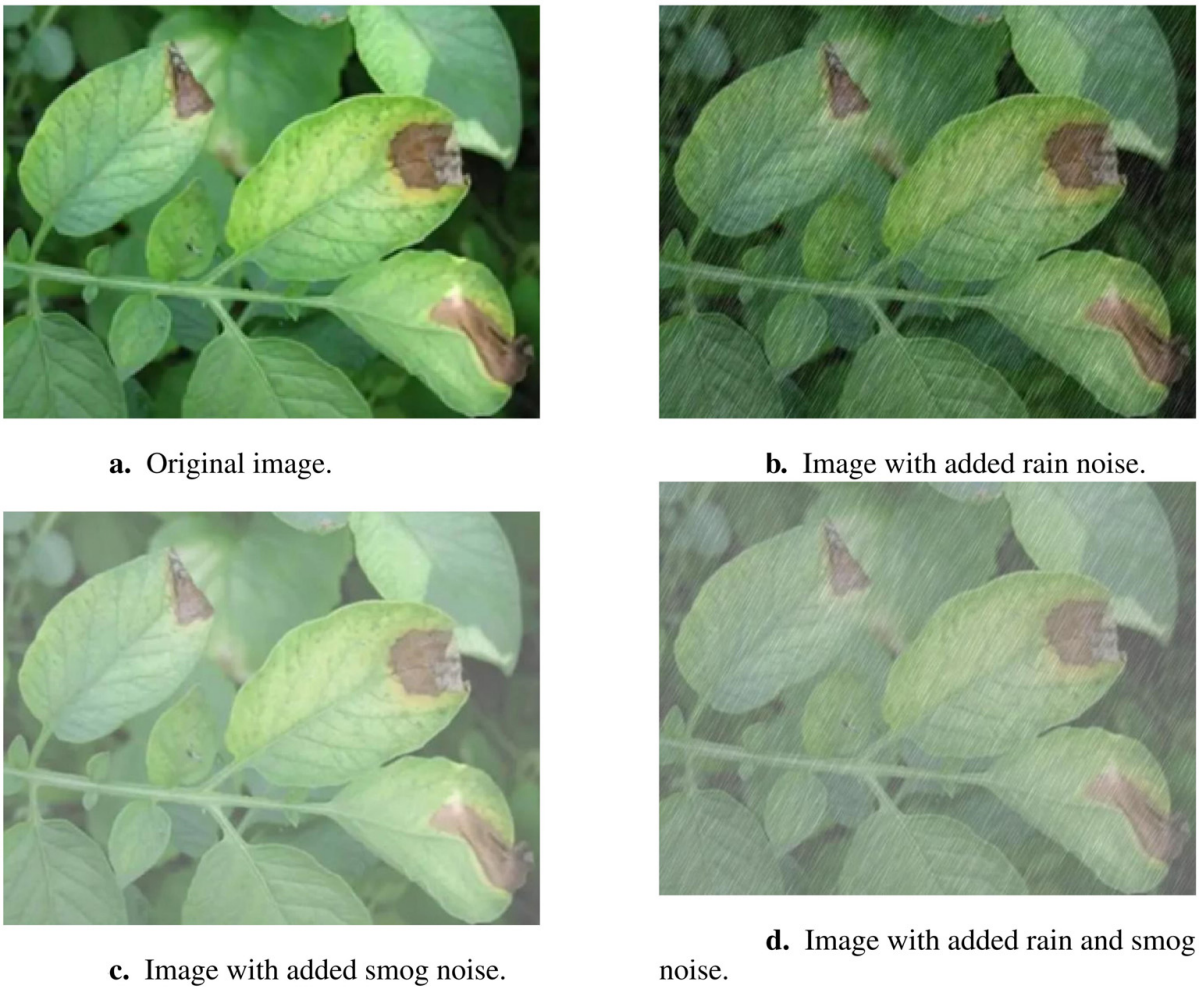
Example images demonstrating the impact of different types of noise added to the original image. **(A)** Original image. **(B)** Image with added rain noise. **(C)** Image with added smog noise. **(D)** Image with added rain and smog noise.

In the data processing stage, various data augmentation strategies were implemented to enhance the model’s generalization and robustness. A Python program utilizing the OpenCV library was developed to artificially add complex rainy and smoggy backgrounds to the images. This augmentation was randomly applied to the dataset images, simulating agricultural climate noise such as rain and smog. These adverse weather conditions are commonly encountered in real-world agricultural settings and can obscure disease symptoms, making it difficult for traditional models to detect plant diseases under such conditions. By introducing this type of noise, the model was trained to better adapt to these challenging environments, improving its ability to identify diseases like early and late blight, even under complex climatic conditions. **Figure 2** illustrates the modifications introduced by the agricultural climate noise, including rain and smog.

Additionally, the dataset was processed using various image enhancement techniques, such as stretching, scaling, and transformations within the HSV color space. Stretching and scaling were applied to simulate varying sizes and proportions of disease symptoms that may occur due to different growth stages of the plants in the field. These treatments help the model learn to recognize disease features at different scales, which is particularly important since plant diseases can manifest differently based on plant size and maturity. Meanwhile, transformations in the HSV color space were used to account for lighting variations, such as shadows or overexposure, that are common in outdoor agricultural environments. These adjustments improve the model’s robustness in detecting diseases regardless of fluctuating field lighting conditions.

Furthermore, image transformation methods such as rotation, flipping, and translation were incorporated to further enrich the dataset’s diversity and complexity. These transformations mimic the natural variability in the orientation and positioning of plants in the field, ensuring the model can generalize across different perspectives and viewing angles, which is crucial when diagnosing diseases in a wide range of real-world settings.

After applying these augmentation techniques, the dataset was significantly expanded. In the augmented training set, there were 785 images labeled with early blight, 805 images labeled with healthy plants, and 850 images labeled with late blight. For the augmented validation set, the counts were 831 images for early blight, 800 images for healthy plants, and 827 images for late blight.

### 2.2 Methods

**Figure 3:**
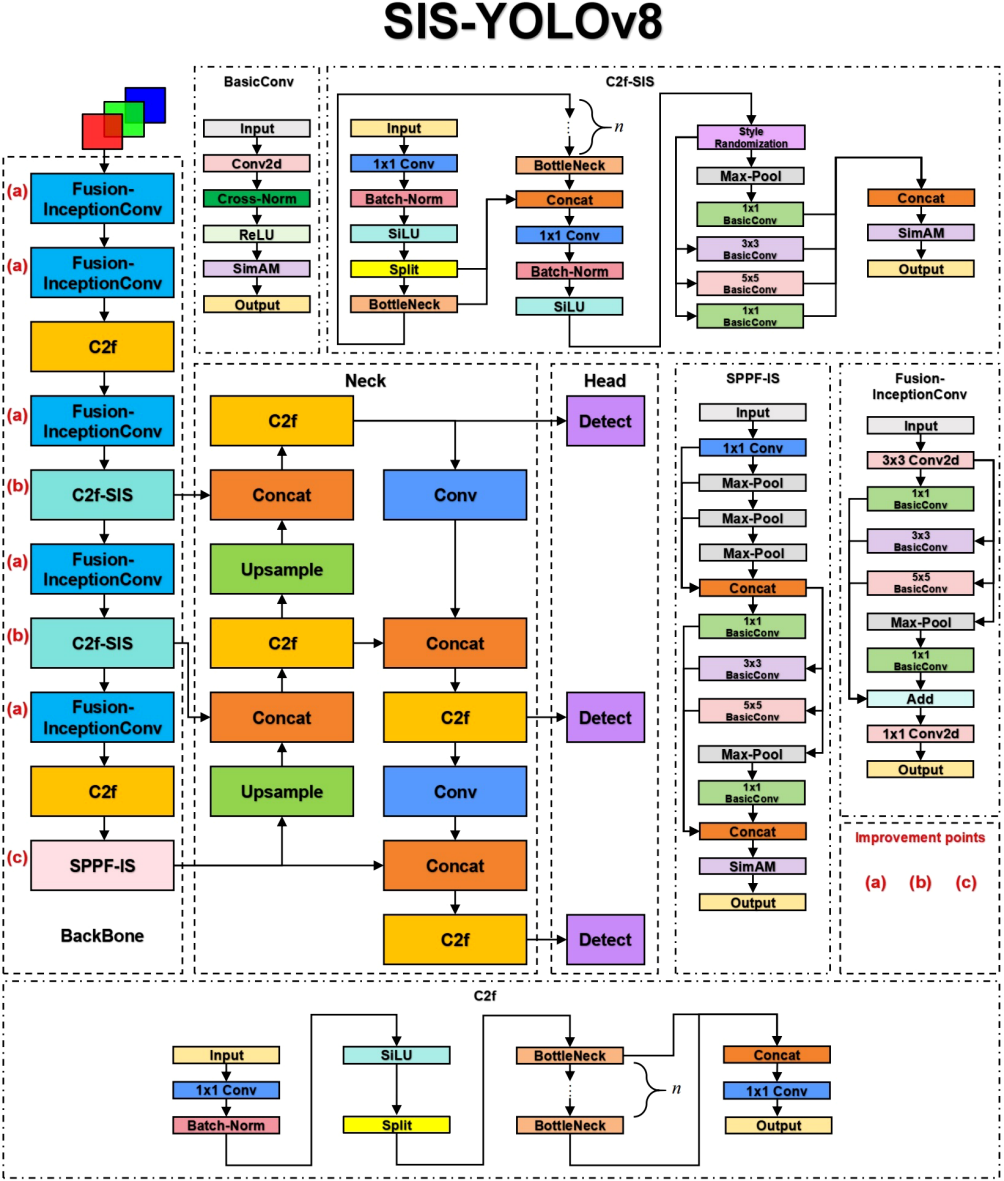
The structure of SIS-YOLOv8.

The YOLOv8n model consists of three primary components: Backbone, Neck, and Head. The Backbone serves as the core network, primarily comprising the Conv module, C2f, and SPPF modules, responsible for feature extraction. The Neck component effectively integrates features of various scales learned by the model. The Head network is responsible for predictions. This model has shown outstanding performance in detecting common diseases in potatoes and tomatoes. This paper presents improvements to the original model, renaming it SIS-YOLOv8 to reflect these enhancements. **Figure 3** illustrates the network architecture of this model.

In this study, the C2f-SIS module was introduced to replace the C2f layer in the YOLOv8n model’s backbone. This modification aims to enhance the model’s domain generalization capabilities and robustness, facilitating multi-scale feature fusion and the decoupling of more detailed features. Furthermore, the Fusion-InceptionConv module was proposed to substitute the Conv layer in the YOLOv8n model’s backbone. This change improves the model’s ability to discern background noise in complex agricultural settings, particularly under challenging weather conditions such as rain and smog. Additionally, this module enables the extraction of more precise textural details from images of diseased leaves. The study also introduces the SPPF-IS module, which enhances feature integration within the feature pyramid framework, allowing for the extraction of even finer-grained features.

#### 2.2.1 BasicConv

Convolution is a fundamental operation in Convolutional Neural Networks (CNNs), a class of deep learning models specifically designed to process grid-like data, such as images. In CNNs, the convolution operation involves applying a small filter (or kernel) to the input image in a sliding window fashion, extracting local features such as edges, textures, and patterns. At each position, the filter performs element-wise multiplication with the image and then sums the results, producing a feature map that highlights the presence of specific patterns. This enables CNNs to automatically learn hierarchical features from raw pixel data, reducing the need for manual feature engineering.

The convolutional layers in CNNs are structured to learn increasingly abstract features at various levels of granularity. The initial layers capture low-level features like edges, while deeper layers combine these low-level features to recognize more complex patterns or objects. This hierarchical approach to feature extraction makes CNNs particularly effective for tasks such as image classification, object detection, and segmentation.

In this study, a novel convolutional architecture named BasicConv2D is proposed, designed to replace the convolutional segment of the enhanced InceptionV1 (Szegedy et al., 2015) structure. This innovative design aims to boost the model’s feature extraction capabilities and robustness, particularly for disease detection tasks in complex agricultural environments. **Figure 3** depicts the structure.

The BasicConv2D architecture consists of a standard 2D convolutional layer (Conv-2D), primarily responsible for initial feature extraction. Following this, the Cross-Norm method is introduced, which significantly improves the model’s generalization capability, especially with cross-domain data. Cross-Norm implements normalization across the channel dimensions of the feature maps, facilitating improved feature distribution and enhancing the model’s consistency throughout the training and testing phases.

After Cross-Norm, the ReLU activation function is incorporated to introduce non-linear properties, thus amplifying the model’s expressive potential. Lastly, the SimAM attention mechanism (Yang et al., 2021) is integrated, an adaptive approach that accentuates meaningful features within the feature maps across both channel and spatial dimensions, while concurrently suppressing irrelevant features. This mechanism adaptively focuses on crucial areas within the image, markedly enhancing the model’s contextual comprehension in agricultural scenarios characterized by fog and rain, thus bolstering the model’s robustness. Through the innovative design of the BasicConv2D structure, not only efficient feature extraction is achieved but also the model’s generalization capabilities and robustness are significantly enhanced by integrating both Cross-Norm and the SimAM attention mechanism.

#### 2.2.2 SimAM attention mechanism

In deep learning, attention mechanisms are inspired by the human visual and cognitive systems. Just as we instinctively focus our attention on important objects in daily life while ignoring irrelevant details, attention mechanisms enable models to prioritize key aspects when processing information, while disregarding unrelated or insignificant parts. This mechanism has demonstrated significant advantages in tasks such as image processing and natural language processing, as it allows models to utilize limited computational resources more efficiently, thereby enhancing performance.

By assigning different “weights” or “attentions” to various features, the attention mechanism helps the model capture more complex and nuanced patterns, thereby improving its learning capacity and decision-making accuracy.

To enhance the model’s ability to distinguish between significant and insignificant features, and to improve its feature extraction capabilities in complex agricultural settings, the SimAM attention mechanism has been incorporated. Drawing on principles from neuroscience, the SimAM mechanism assesses the importance of each neuron, thereby enhancing meaningful features within the feature map while suppressing irrelevant ones. This method’s essence is to emulate the variability of neuronal firing modes and their mutually suppressive interactions in the spatial domain.

**Figure 4:**
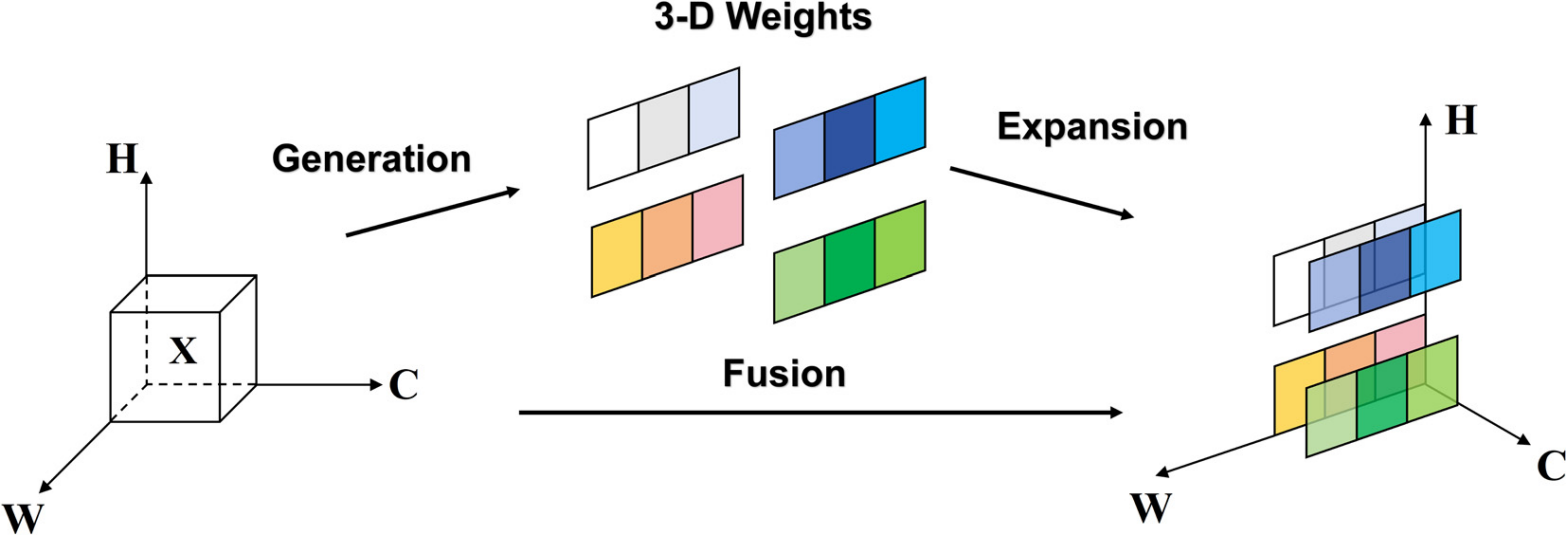
Full 3-D weights for SimAM attention.

As depicted in **Figure 4**, the SimAM attention mechanism is a 3D attention mechanism, distinct from traditional channel and spatial attention mechanisms. It integrates these two approaches. Within 3D feature maps, adjacent pixels typically demonstrate strong similarities, in contrast to the weaker similarities among more distantly located pixels. SimAM capitalizes on this trait by calculating the similarity between each pixel and its adjacent counterparts to generate attention weights.

The SimAM attention mechanism quantifies the linear separability between the target neuron and other neurons, utilizing this metric to evaluate the significance of neurons. This method seeks to pinpoint neurons that are rich in information and demonstrate unique firing patterns, consequently attributing greater importance to them. By doing so, the model adaptively concentrates on crucial areas within the feature map, markedly improving the model’s contextual comprehension during disease detection tasks under agricultural conditions impacted by haze and rain, thereby enhancing its robustness.

Moreover, the SimAM attention mechanism bolsters model robustness by exerting spatial suppression effects via the inhibition of surrounding neurons. This spatial suppression aids the model in more effectively differentiating between essential and non-essential features, thereby facilitating more precise disease detection in intricate agricultural environments.

The formula for the SimAM neuronal energy function is as follows.


(1) $$e_{t}^{*}=\frac{4\left(\hat{\sigma^{2}}+\text{λ}\right)}{{\left(t-\hat{u}\right)}^{2}+2{\hat{\sigma }}^{2}+2\text{λ}}$$

In this context, *µ* denotes the mean of the input feature map across dimensions *H* and *W*, whereas *σ* represents the standard deviation of the input feature map along these same dimensions. The parameter λ generally functions as a scaling factor, employed to modulate the response intensity of the energy function to the features. The lower the energy, the more pronounced the distinction between neuron *t* and its adjacent neurons, thereby increasing its significance. As a result, the importance of a neuron can be ascertained through $\frac{1}{e^{*}}$ .

#### 2.2.3 Design of C2f-SIS module

As shown in **Figure 3**, the C2f-SIS module enhances the model’s generalization power and robustness. This module builds upon the foundational YOLOv8 C2f module and incorporates significant improvements through an effective enhancement module. The C2f-SIS plays a crucial role in advancing the domain generalization capabilities within the SIS-YOLOv8 framework.

To address the model’s low domain generalization ability, especially in recognizing identical disease features across various crops, the Style Randomization module was incorporated into the C2f-SIS. This technique aims to boost the model’s robustness against unseen domains by converting the features of original samples into new, randomly styled features. Using an encoder-decoder network, this method integrates noise information with original style data within a hidden space. This approach offers more precision than traditional image-based data augmentation methods and is more suitable for tackling domain generalization issues.

For any given input training image *x*, the feature map of *x* is defined as $f_{x}\in {\mathbb{R}}^{C\times H\times W}$ , where *H* and *W* represent the spatial dimensions, and *C* indicates the number of channels. Consequently, the formula for IN is presented as follows.


(2) $$\text{IN }\left(f_{x}\right)=\text{γ}\frac{f_{x}-f_{\mu }}{f_{\sigma }}+\beta$$

In the formula, $\text{γ},\beta \in {\mathbb{R}}^{C}$ are learnable affine transformation parameters, and $f_{\mu },f_{\sigma }\in {\mathbb{R}}^{C}$ represent the mean and standard deviation of each feature map channel, respectively. Their formulas are as follows.


(3) $$f_{\mu }=\frac{1}{HW}\sum_{h=1}^{H}\sum_{w=1}^{W}f_{clw}$$

(4) $$f_{\sigma }=\sqrt{\frac{1}{HW}\sum_{h=1}^{H}\sum_{w=1}^{W}{\left(f_{chw}-f_{\mu }\right)}^{2}+\epsilon }$$

Herein, ϵ is a constant used for numerical stability. It achieves the randomization of feature map styles by randomly perturbing the mean and standard deviation of each feature map channel. Additionally, it uses the random style statistics generated by AdaIN to replace the original feature maps. The formula is as follows.


(5) $$\text{AdaIN }\left(f_{x},s\right)=s_{\sigma }\frac{f_{x}-f_{\mu }}{f_{\sigma }}+s_{\mu }$$

**Figure 5:**
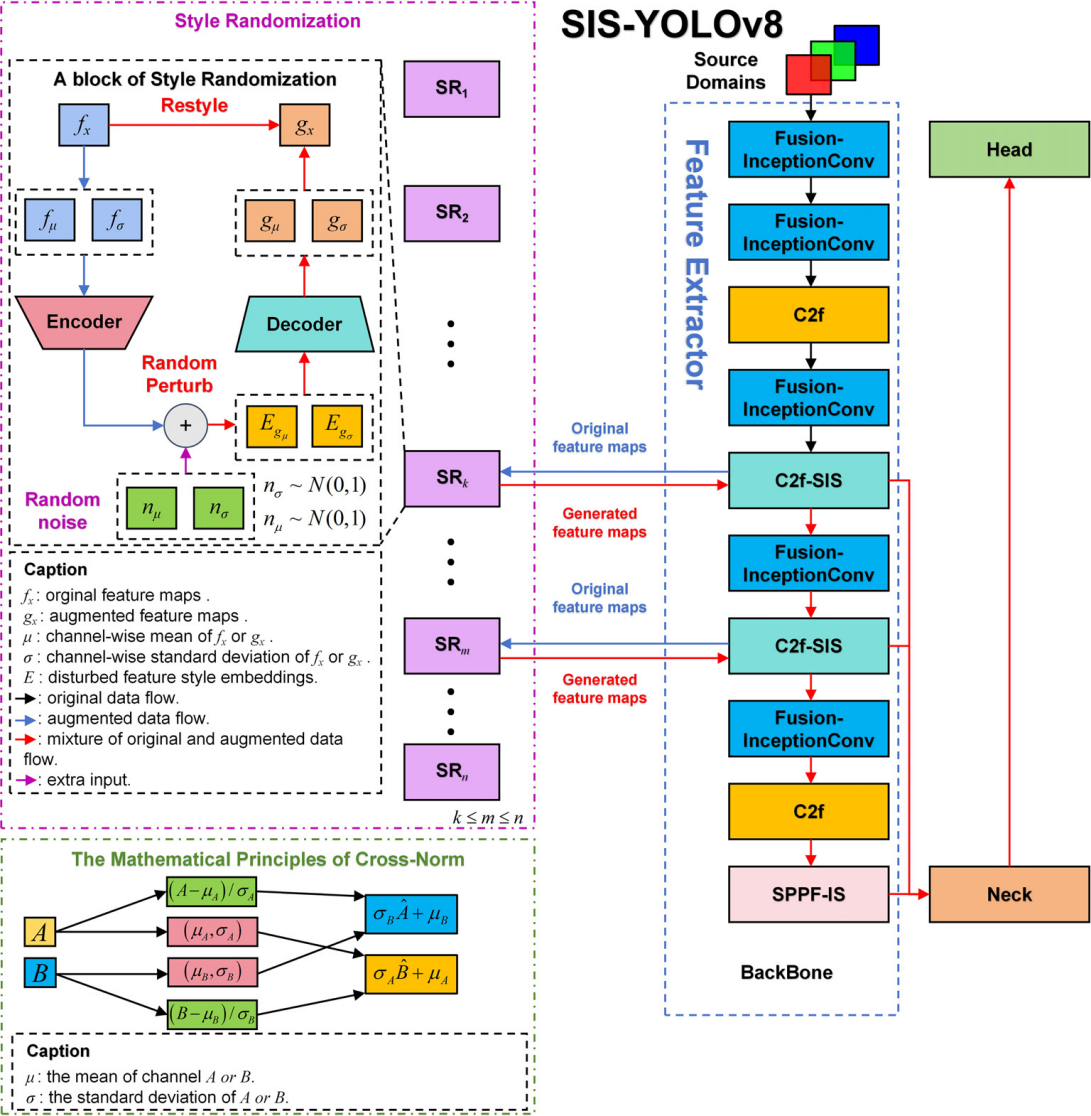
An overview of style randomization and cross-norm in SIS-YOLOv8, in this study, the second and third C2f layers of the original YOLOv8n are replaced with C2f-SIS, and the generalization ability of the model is enhanced by incorporating C2f-SIS into the middle of the backbone network.

**Figure 5** illustrates the module used for domain generalization within the C2f-SIS framework. The network architecture of Style Randomization, depicted in **Figure 5**, facilitates random style transformations by adding noise to the feature maps. Operating within the feature space, this module enables diverse and abstract transformations of input images. Thus, compared to traditional image-based enhancements, this approach allows the enhanced features to encompass a broader spectrum of possible styles and distributions.

In the Style Randomization process, the variable (K) represents the number of source domains, with each source domain allocated a distinct Style Randomization module for generating various styles. For instance, in the backbone network of SIS-YOLOv8, as shown in **Figure 3**, each C2f-SIS layer incorporates a Style Randomization module (SR module). This module enriches the original feature maps through the addition of random noise, resulting in more stylized feature representations.

To enhance the domain generalization capabilities of the model, Cross-Norm has been implemented, replacing traditional normalization techniques. Unlike conventional methods such as Batch-Norm and Layer-Norm, which standardize feature maps but do not address style variability, Cross-Norm introduces a style transfer mechanism that increases the diversity of the training data. This approach helps to mitigate differences in data distribution between training and testing datasets, thereby improving the model’s generalization ability. Traditional normalization methods, which overlook style variations, can lead to suboptimal model performance on datasets with diverse distributions. Typically, normalization techniques assume homogeneity in data distribution between training and testing phases; however, variations in data distribution are common in practical scenarios. As illustrated in **Figure 3**, this paper introduces Cross-Norm as the normalization layer within BasicConv to enhance the model’s generalization power and robustness in the face of significant distribution changes.

As depicted in **Figure 5**, Cross-Norm boosts the model’s generalization capacity across various styles of feature maps by exchanging the means and standard deviations between two distinct channels of the feature maps. Specifically, if Channel A has a mean of $\mu_{A}$ and a standard deviation of $\sigma_{A}$ , and Channel B has a mean of $\mu_{ℬ}$ and a standard deviation of $\sigma_{ℬ}$ , then the Cross-Norm normalization formula for these channels is defined as follows.


(6) $$\sigma_{ℬ}\frac{A-\mu_{A}}{\sigma_{A}}+\mu_{ℬ},\;\sigma_{A}\frac{ℬ-\mu_{ℬ}}{\sigma_{ℬ}}+\mu_{A}$$

#### 2.2.4 Design of Fusion-InceptionConv module

To enhance feature extraction robustness in complex environments such as rain and haze, this study introduces the Fusion-InceptionConv module, depicted in **Figure 3**. This module enhances the Conv module from the YOLOv8n model by incorporating elements from the InceptionV1 architecture. The Fusion-InceptionConv module consists of a 3x3 convolutional layer followed by a modified branch structure, named InceptionV1+. The 3x3 convolutional layer is designed to capture local features of the input image, while the InceptionV1+ branch structure is aimed at exploring and integrating global features. The InceptionV1+ structure includes multiple parallel paths, each equipped with various convolutional layers, such as 1x1, 3x3, and 5x5, alongside pooling layers. These parallel paths enable the module to capture and amalgamate features across different spatial resolutions and extents, thereby offering a more comprehensive representation of the input image.

Following the InceptionV1+ branch structure, the outputs of each path are concatenated and then passed through a 1x1 convolutional layer. This final layer is responsible for fusing the diverse features captured by the InceptionV1+ branches and reducing the feature dimensions, effectively representing and classifying the input image.

Moreover, the original concatenation operation in the InceptionV1 branch structure is replaced with an addition operation in the InceptionV1+ to enhance feature fusion. This modification aims to improve fusion capability through element-wise addition, rather than mere concatenation, thus better preserving the independence of features from each branch and facilitating effective integration at the element level. This improvement significantly enhances the model’s robustness and generalization capability in detecting diseases under complex agricultural climatic conditions.

#### 2.2.5 Design of SPPF-IS module

This study introduces an enhanced version of the original SPPF module from the YOLOv8n model, named SPPF-IS, which incorporates the InceptionV1+ architecture and the SimAM attention mechanism, as illustrated in **Figure 3**. The SPPF-IS module leverages the principles of the Feature Pyramid Network, integrating the InceptionV1+ module with the SimAM attention mechanism to emphasize critical feature regions and enhance feature discriminability. This architecture allows our model to effectively amalgamate multi-scale features and employ the attention mechanism to adaptively modulate the feature map weights, thereby significantly boosting the model’s capability to handle complex backgrounds and improve detection accuracy.

#### 2.2.6 DepGraph pruning

DepGraph is a general-purpose, automated structured pruning method. The primary aim of this method is to streamline large neural network architectures, such as CNNs, Transformers, RNNs, GNNs, etc. In deep neural networks, the parameters of different layers are inherently interdependent within the network architecture. By reducing the model’s complexity, pruning significantly improves its inference speed, allowing for faster processing and higher FPS metrics. This enhancement is crucial for real-time agricultural applications, such as rapid disease detection in the field, where timely responses are essential for effective intervention and monitoring. DepGraph facilitates pruning by automatically analyzing the complex structural dependencies between network layers and comprehensively grouping coupled parameters. The formula for layer dependency relationships in deep neural networks is as follows:


(7) $$(f_{1}^{-},\underset{\text{Inter}-\text{layer Dep}}{\underset{︸}{f_{1}^{+})↔(f_{2}^{-}}},f_{2}^{+})\cdots ↔\underset{\text{Intra}-\text{layer Dep}}{\underset{︸}{\left(f_{L}^{-},f_{L}^{+}\right)}}$$

**Figure 6:**
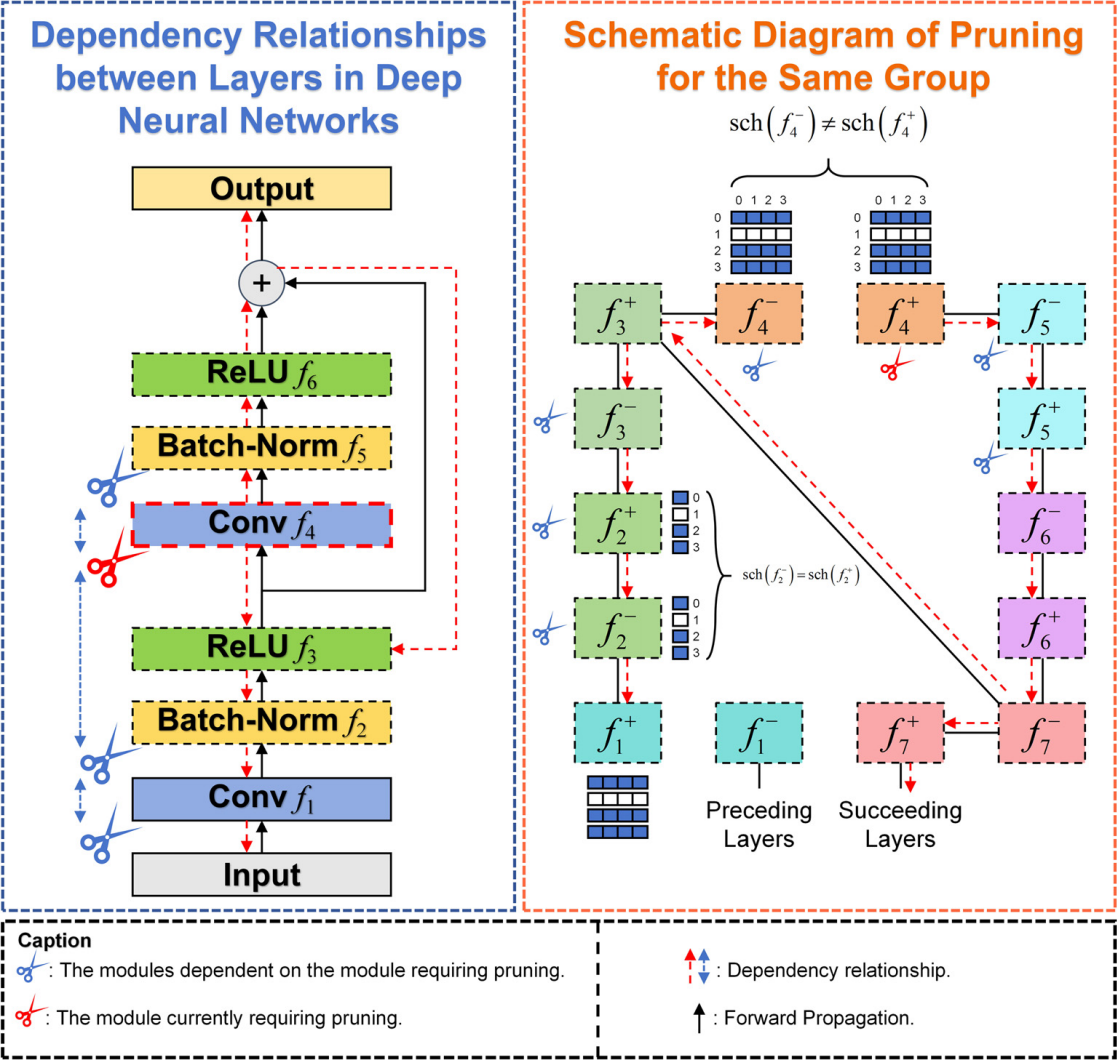
DepGraph pruning.

Where the symbol ↔ denotes the connection between two adjacent layers and the interlayer dependency can be represented as $f_{i}^{-}↔f_{j}^{+}$ . The pruning dependency relationship is illustrated in **Figure 6**, if pruning $\text{Conv }f_{4}$ , then all other dependent layers $f_{5},f_{2},f_{1}$ must be pruned simultaneously.

DepGraph Pruning algorithm recursively deduces the required grouping matrix **G** using the local dependency relationships between adjacent layers, pruning redundant parameters within the same group. Different groups can be pruned independently. This local dependency relationship between adjacent layers is referred to as a Dependency Graph (DG). According to the grouping matrix **G**, the Dependency Graph **D** is obtained, and direct connectivity layers are modeled for dependency. Therefore, the grouping problem can be simplified into a path search problem. When there is a path between node i and node j in Dependency Graph D, it indicates that the current node belongs to the same group. As shown in **Figure 6**, layers in the same group adopt the same grouping scheme, i.e., $\text{sch }\left(f_{i}^{-}\right)=\text{sch }\left(f_{i}^{+}\right)$ .

## 3 Results

### 3.1 Performance metrics for network models

This study employs Precision (P), Recall (R), Mean Average Precision (mAP) as evaluation metrics for the experiments. The formulas used to calculate these metrics are as follows:


(8) $$\text{ Precision }=\frac{TP}{TP+FP}\times 100\%$$

(9) $$\text{ Recall }=\frac{TP}{TP+FN}\times 100\%$$

(10) $$\text{AP}=\int_{0}^{1}P\left(R\right)dR\times 100\%$$

(11) $$\text{mAP}=\frac{\sum_{1}^{N}\int_{0}^{1}P\left(R\right)dR}{N}\times 100\%$$

### 3.2 Experiment environments

This experiment was conducted using the PyTorch deep learning framework and a Windows 10 Professional system. The CPU is 12th Gen Intel(R) Core(TM) i9-12900HX 2.30 GHz, the GPU is NVIDIA GeForce RTX 3080Ti Laptop, and the memory is 64GB. The program was written using CUDA 11.8 and Python 3.9.

### 3.3 Hyperparameter tuning experiment

#### 3.3.1 The impact of K value in Style Randomization on the model

This experiment investigated the influence of the K value in the Style Randomization module on the accuracy of a model equipped with a C2f-SIS module in its backbone network, but lacking Fusion-InceptionConv and SPPF-IS modules. The K value was varied from 1 to 10, and the results are presented in **Table 1**. The study highlights the role of the Style Randomization module in boosting the generalization capabilities of the C2f layer within the YOLOv8n model by adjusting the K parameter, which controls the intensity of style randomization. The experimental findings indicate that the model performs optimally across various evaluation metrics when K is set to 1. This outcome suggests that even minimal style randomization, by introducing random noise and creating new features with varied styles, can significantly enhance model generalization. Thus, Style Randomization proves to be a potent method for improving generalization in object detection tasks, enabling the model to better handle the diversity and complexity of real-world scenarios.

**Table 1:** Experimental results of style randomization’s K value.

K	Precision(%)	Recall(%)	mAP50(%)	mAP50-95(%)
**1**	**83.9**	**79.8**	**85.9**	**82.2**
2	81.0	79.3	84.6	81.4
3	83.7	80.8	85.5	82.2
4	79.8	75.9	82.2	78.6
5	81.1	79.5	84.6	81.4
6	79.7	77.0	82.1	79.2
7	82.1	76.8	84.1	80.3
8	84.1	75.3	84.5	80.2
9	84.5	79.1	85.7	82.1
10	80.3	78.2	84.0	81.0

The bold values represent the optimal parameters for each experiment.

### 3.4 Ablation experiment

#### 3.4.1 Module ablation experiment

The ablation study results are summarized in **Table 2**. In Experiment 2, incorporating only the Fusion-InceptionConv module led to a 3.8% increase in accuracy, a 2.8% decrease in recall, a 2.5% increase in mAP50, a 3.1% rise in mAP50-95, and a 3.23M increase in parameter size. These results demonstrate that the Fusion-InceptionConv module enhances the model’s feature fusion capabilities across various scales, improving feature extraction even in complex agricultural climatic conditions like rain and smog. In Experiment 3, setting the K value of Style Randomization in the C2f-SIS layer to 1 after introducing only the C2f-SIS module resulted in a 5.5% improvement in accuracy, a 2% increase in recall, a 3.4% rise in both mAP50 and mAP50-95, and a 0.18M increase in parameters. These findings underscore the C2f-SIS module’s efficacy in boosting domain generalization, allowing for better differentiation of disease features across multiple scales and enhancing detection capabilities across different species afflicted with the same disease. Experiment 4 explored the impact of incorporating only the SPPF-IS module, which led to a 2.1% decrease in accuracy, a 4% reduction in recall, a 1.7% drop in mAP50, a 1.3% decrease in mAP50-95, and a 0.59M increase in parameter size. This experiment shows that the SPPF-IS module can further enhance feature fusion based on the pyramid structure, improving the model’s representation of complex, fine-grained features. Experiment 8, which integrated all three modules, not only improved the foundational performance of the model but also its domain generalization and feature decoupling capabilities under complex agricultural weather conditions. This integration led to a 6.4% improvement in accuracy, a 4.5% increase in recall, a 5.8% rise in mAP50, and a 5.7% enhancement in mAP50-95, with a parameter increase of 4 million. Despite the significant increase in parameters, all performance metrics reached their peak values, indicating a strong overall enhancement. Given the substantial rise in parameter count, future studies will focus on conducting pruning experiments on the model.

**Table 2:** Modules ablation study: Here, ‘a’ corresponds to the Fusion-InceptionConv module, ‘b’ to the C2f-SIS module, and ‘c’ to the SPPF-IS module.

No.	a	b	c	Precision(%)	Recall(%)	mAP50(%)	mAP50-95(%)	Params(M)
1	×	×	×	77.5	78.2	81.6	77.7	3.01
2	✓	×	×	81.3	75.4	84	80.8	6.24
3	×	✓	×	83.9	79.8	85.9	82.2	3.19
4	×	×	✓	75.4	74.2	79.9	76.4	3.6
5	✓	✓	×	83.2	79.6	85.7	82	6.42
6	✓	×	✓	80.3	74.7	82.2	79	6.83
7	×	✓	✓	80.6	79	83.1	79.3	3.64
**8**	✓	✓	✓	**84.1**	**82.8**	**87.4**	**83.4**	**7.01**

The symbol "✓" indicates that the corresponding module is included in the ablation study, while the symbol "×" indicates that the corresponding module is excluded, with bold values representing the best performance achieved in each case.

### 3.5 Pruning experiments

The DepGraph pruning method is used to prune the SIS-YOLOv8 model. The pruning experiment consists of three main stages:sparse training, model pruning and model fine-tuning.

#### 3.5.1 Sparse training experiment

Sparse training achieves sparsity by adding L1 regularization to the γ parameter of Batch Normalization (BN) in the loss function, causing the γ values corresponding to the majority of channels to approach 0, thus enabling the model to achieve a sparse effect. The equation of L1 regularization is shown as follows:


(12) $$L_{L1}\left(\text{γ}\right)=L\left(w\right)+\text{λ}\left|\text{γ}\right|$$

Where *ω* are the model’s parameters. In this study, sparse training was conducted for 500 epochs to ensure the convergence of the sparse training process. The pruning rate was set at 0.5, as determined from the experiments shown in **Table 3**, which indicate that a pruning rate of 0.5 yields the best performance. As shown in **Table 3**, the model’s training loss varied as the pruning rate was adjusted, and the optimal pruning rate was identified to balance sparsity and model accuracy. The value of γ was set to the default parameter of the DepGraph model.

**Table 3:** Pruning rate experiments.

Pruning Rate	Precision(%)	Recall(%)	mAP50(%)	mAP50-95(%)	Params/(M)
0.25	59.3	59.3	58.3	24.6	1.05
**0.5**	**85.7**	**82.2**	**87.5**	**84**	**2.41**
0.75	79.5	79.6	84.6	81.6	5.36

The bold values correspond to the optimal pruning rates achieved for each model configuration.

#### 3.5.2 Model pruning experiment

Model pruning is a technique that reduces the size and computational load of a model by eliminating redundant parameters. This process typically involves identifying and removing parameters that contribute less to the task using pruning algorithms. The primary objective of model pruning is to decrease the number of parameters and computational complexity while maintaining the model’s performance. In this study, a pruning ratio of 0.5 was selected, resulting in the removal of half of the model’s parameters. Finetuning was subsequently performed on this pruned model. **Table 4** presents the metrics of the SIS-YOLOv8 model after sparse training and model pruning on the datasets used in this study.

**Table 4:** Precision after model pruning.

Evaluation Indicators	Value
Precision(%)	78.0%
Recall(%)	79.0%
mAP50(%)	84.4%
mAP50-95(%)	80.8%

#### 3.5.3 Model finetune experiment

Following the model pruning, a fine-tuning process was conducted. Fine-tuning involves further adjustments and optimizations to the pruned model to restore or enhance its performance. This step is crucial to ensure that pruning does not lead to performance degradation.

The fine-tuning training parameters were set as follows:

The image input size was 640x640 pixels. The training was conducted for 70 epochs to allow the model sufficient iterations for convergence. The learning rate was set at 0.01, using the Stochastic Gradient Descent (SGD) optimizer. The Intersection over Union (IoU) threshold was 0.7, momentum was set to 0.937, and weight decay was 0.0005. Except for the number of epochs, these parameters are the default values specified in the YOLOv8 framework, which simplifies the process of parameter tuning for consistent and reproducible results.

**Table 5** presents the results of the pruned SIS-YOLOv8 model. Compared to the pre-pruned model, the pruned version showed a reduction of 4.6 million parameters and an increase in accuracy by 1.6%. However, there was a slight decrease in the recall rate by 0.6%. The mAP50 improved by 0.1%, and mAP50-95 increased by 0.6%, indicating an enhancement in precision alongside a reduction in parameter count.

**Table 5:** Pruning results of the model.

Model	Precision(%)	Recall(%)	mAP50(%)	mAP50-95(%)	Params/M)
YOLOv8n	77.5	78.2	81.6	77.7	3.01
SIS-YOLOv8	84.1	82.8	87.4	83.4	7.01
**(pruned)SIS-YOLOv8**	**85.7**	**82.2**	**87.5**	**84**	**2.41**

The bold values correspond to the optimal pruning results achieved for the model.

### 3.6 Comparison experiments

Among the various object detection models, SSD (Liu et al., 2016) and FasterRCNN stand out as classic algorithms frequently employed in agricultural disease detection tasks (Fuentes et al., 2017). Similarly, the YOLO series is renowned for its high-speed processing capabilities and treats object detection as a regression issue, predicting bounding boxes and class probabilities in a single network pass. These models are particularly favored in agricultural settings. In this research, the newly developed SIS-YOLOv8 model was compared with SSD-VGG, FasterRCNN-ResNet50, YOLOv5n, YOLOv7-tiny (Wang et al., 2023), YOLOv8n, YOLOv9, YOLOv9-tiny, and YOLOv10-n, as detailed in **Table 6**. The findings reveal that the SIS-YOLOv8 model outperformed the others in all metrics, achieving a mAP50 of 87.4%, mAP50-95 of 83.4%, pruned model mAP50 of 86.5%, and mAP50-95 of 83.5%.

**Table 6:** Comparison experiment results.

Model	Precision(%)	Recall(%)	mAP50(%)	mAP50-95(%)	Params/(M)	FPS
SSD-VGG	75.9	48.9	59.9	38.2	26.3	45.5
FasterRCNN-ResNet50	57.5	46.9	56.4	36.5	137.1	7.5
YOLOv5n	76.0	75.4	78.8	68.9	1.76	301.3
YOLOv7-tiny	72.3	74.0	73.6	63.6	6.02	153.1
YOLOv8n	77.5	78.2	81.6	77.7	3.01	260.1
YOLOv9-tiny	80.3	75.1	81.9	77.9	2.61	277.9
YOLOv9	80.3	77.2	83	79.7	60.5	32.5
YOLOv10-n	78	73.4	78.3	72.8	2.7	283.0
SIS-YOLOv8	84.1	82.8	87.4	83.4	7.01	89.6
**(pruned)SIS-YOLOv8**	**85.7**	**82.2**	**87.5**	**84**	**2.41**	**295.5**

The bold values specifically indicate the optimal results of the comparison experiments.

**Figure 7:**
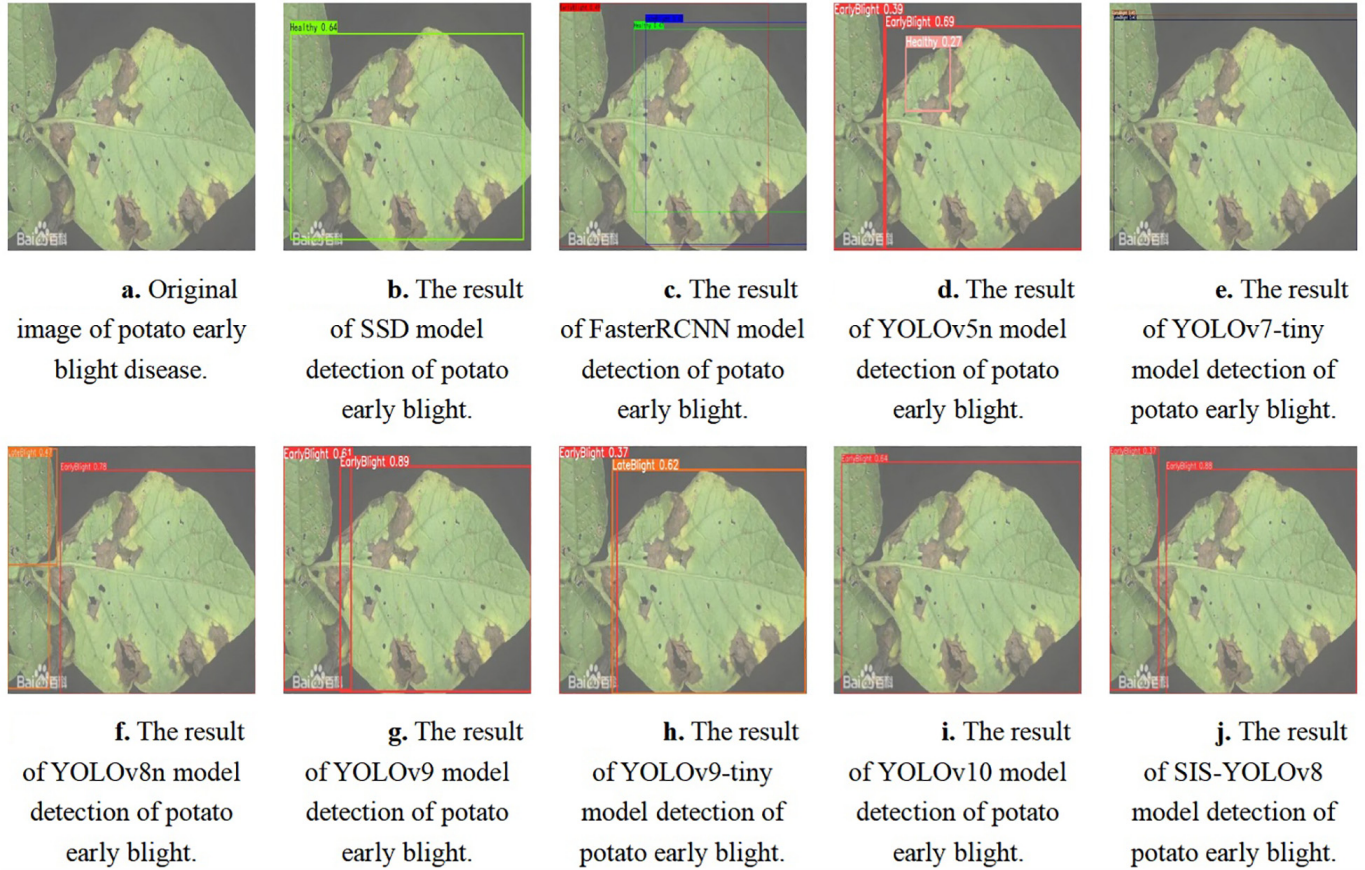
Detection results of potato early blight using different models. **(A)** Original image of potato early blight disease. **(B)** Detection result using the SSD model. **(C)** Detection result using the FasterRCNN model. **(D)** Detection result using the YOLOv5n model. **(E)** Detection result using the YOLOv7-tiny model. **(F)** Detection result using the YOLOv8n model. **(G)** Detection result using the YOLOv9 model. **(H)** Detection result using the YOLOv9-tiny model. **(I)** Detection result using the YOLOv10 model. **(J)** Detection result using the SIS-YOLOv8 model.

**Figure 8:**
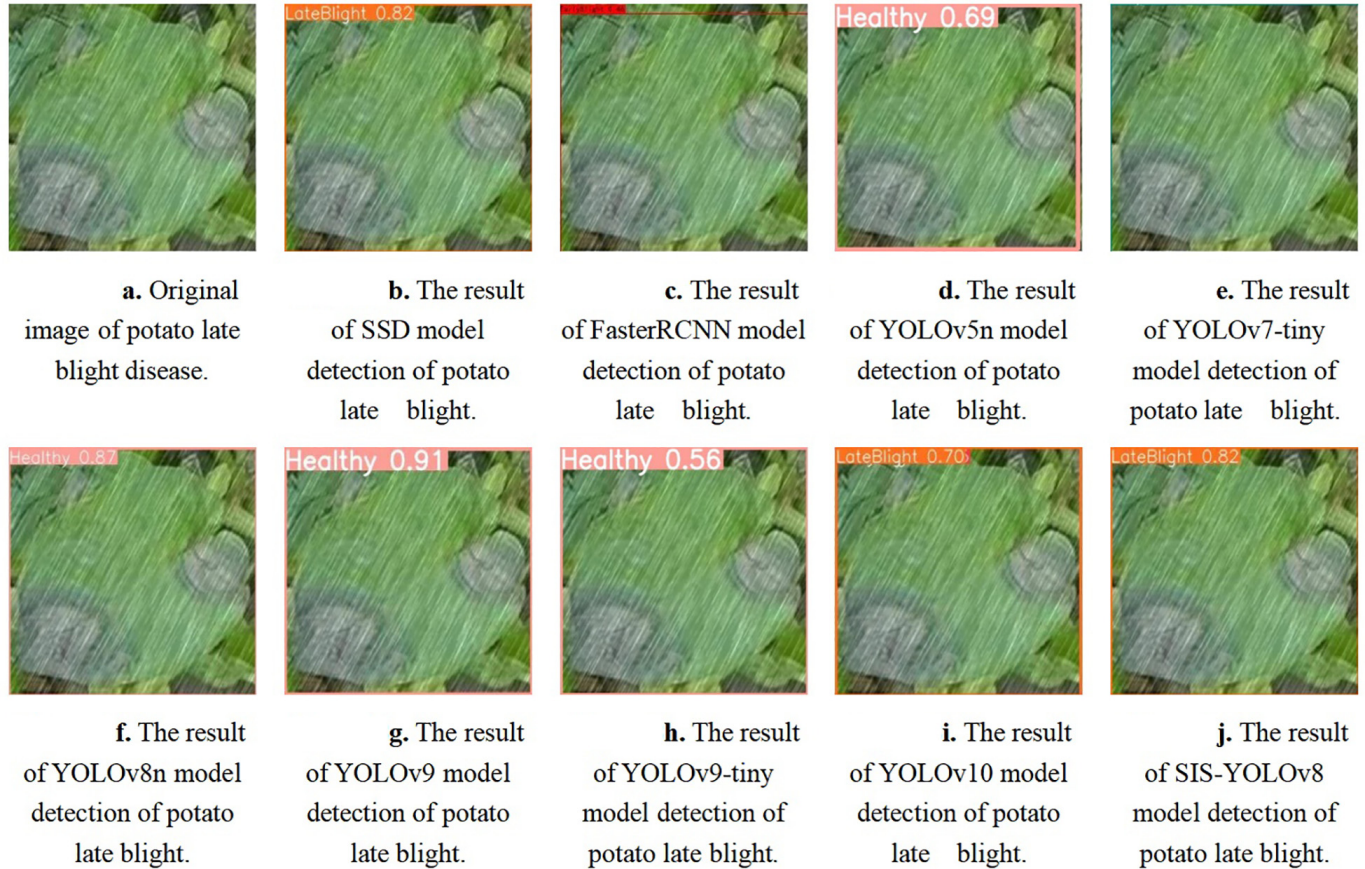
Detection results of potato late blight using different models. **(A)** Original image of potato late blight disease. **(B)** Detection result using the SSD model. **(C)** Detection result using the FasterRCNN model. **(D)** Detection result using the YOLOv5n model. **(E)** Detection result using the YOLOv7-tiny model. **(F)** Detection result using the YOLOv8n model. **(G)** Detection result using the YOLOv9 model. **(H)** Detection result using the YOLOv9-tiny model. **(I)** Detection result using the YOLOv10 model. **(J)** Detection result using the SIS-YOLOv8 model.

**Figure 9:**
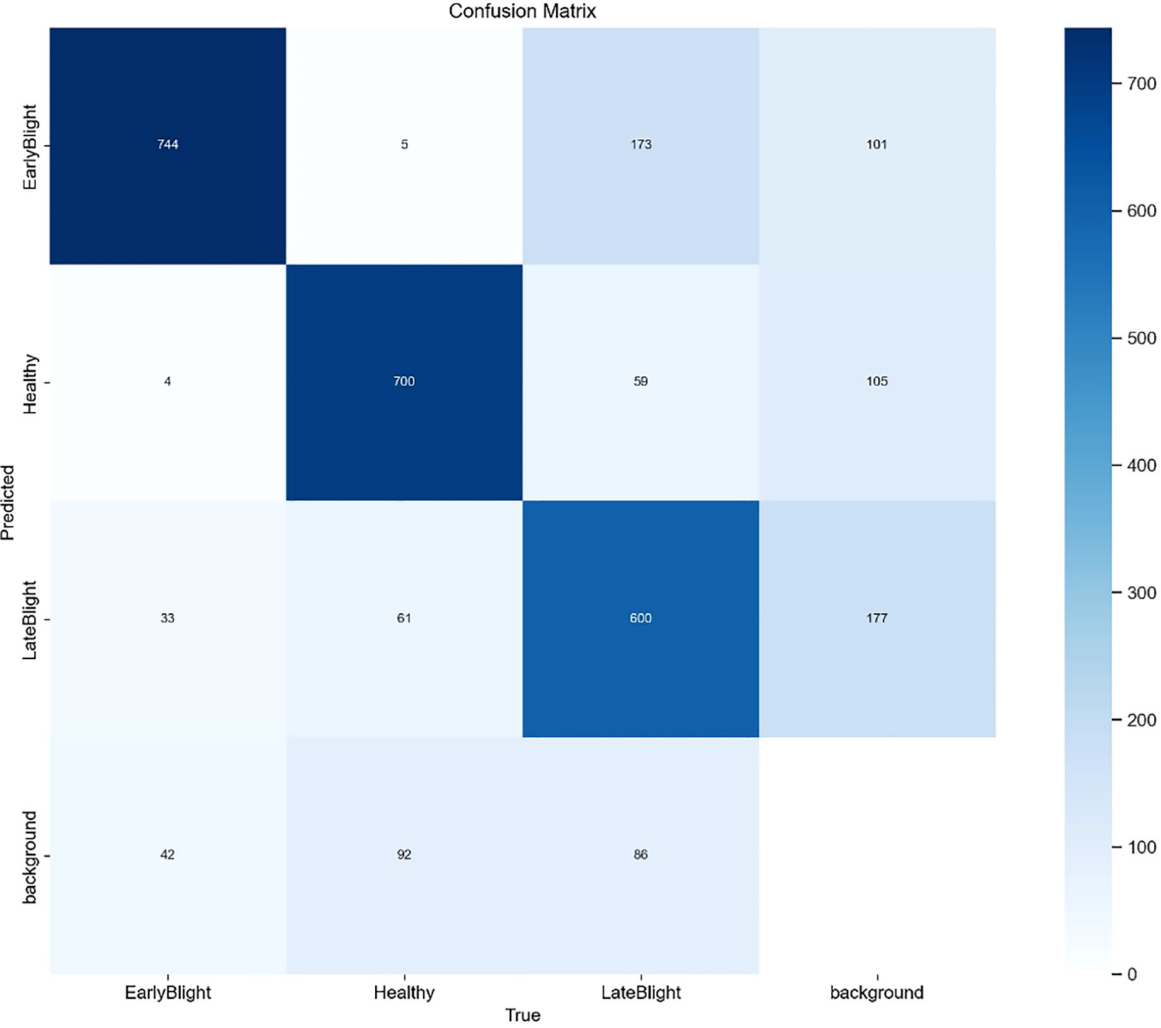
Confusion matrix.

The SIS-YOLOv8 model demonstrated superior performance across all metrics. Although the recall rate of the pruned model decreased by 0.6% compared to its unpruned state, enhancements were observed in all other metrics. Notably, the pruned SIS-YOLOv8 model, with a parameter count similar to YOLOv5n, is only 27% heavier (an increase of 0.65M parameters), and although its FPS is 2% lower than YOLOv5n (a decrease of 5.8 fps), it significantly outperforms YOLOv5n in detection accuracy and robustness. In addition, compared to the unpruned SIS-YOLOv8 model, the pruned version saw an impressive 330% increase in FPS (a boost of 205.9 fps), demonstrating substantial efficiency gains without sacrificing detection performance. To further demonstrate the effectiveness of this improved model in detecting common diseases on potato and tomato leaves, several disease detection images were analyzed, as illustrated in **Figures 7**, **8**. The SIS-YOLOv8 model showed commendable results, effectively completing the task of target detection across various domains under complex climatic conditions, while other models faced challenges with erroneous anchor box detections to varying degrees. Additionally, to better understand the distribution of misclassifications, the confusion matrix of the model’s predictions is shown in **Figure 9**, which highlights the types of errors and the model’s ability to distinguish between different diseases.

## 4 Discussion

**Figure 10:**
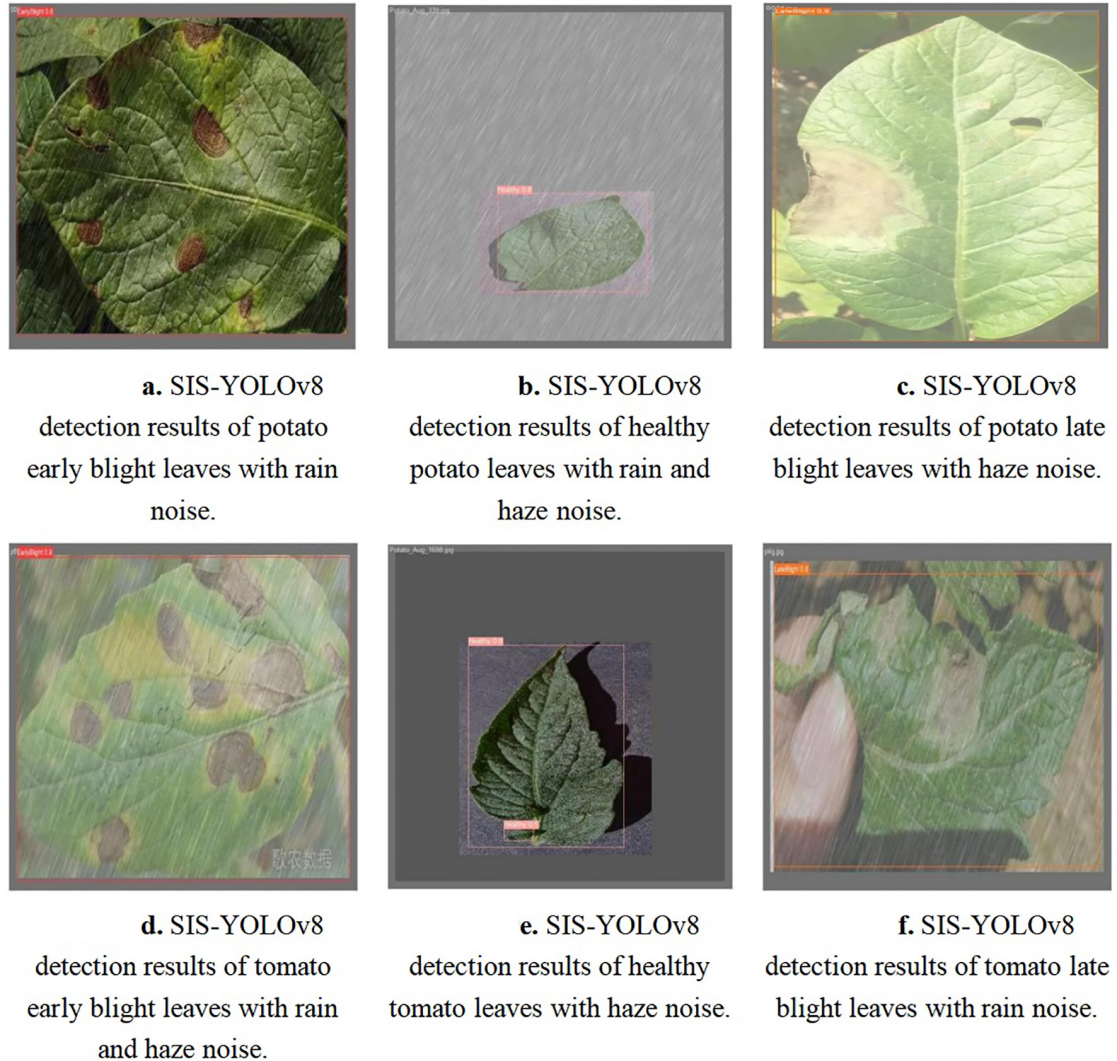
Detection results for early blight, late blight, and healthy leaves in potatoes and tomatoes under complex climate noise using the SIS-YOLOv8 model. **(A)** Detection results of potato early blight leaves with rain noise. **(B)** Detection results of healthy potato leaves with rain and haze noise. **(C)** Detection results of potato late blight leaves with haze noise. **(D)** Detection results of tomato early blight leaves with rain and haze noise. **(E)** Detection results of healthy tomato leaves with haze noise. **(F)** Detection results of tomato late blight leaves with rain noise.

In summary, the SIS-YOLOv8 model presented in this research exhibits high accuracy and effectively addresses the challenge of detecting common diseases in potatoes and tomatoes against complex backgrounds, fulfilling the intended design objectives. Demonstrated in **Figure 10**, the SIS-YOLOv8 model is robust in complex environments, capable of detecting relevant diseases under challenging conditions such as rain and smog. These environmental challenges were simulated by adding noise to the images, mimicking the effects of adverse weather conditions on the quality of visual data. Additionally, the model performs well across domains, transitioning effectively from tomato leaf disease data to potato leaf disease data. The inclusion of the C2f-SIS module yields effective results in cross-domain experiments, using the tomato disease dataset for training and the potato disease dataset for validation, thereby enhancing the model’s robustness and generalization capabilities. The Fusion-InceptionConv module isolates finergrained disease features, enhancing feature extraction capabilities and accuracy under complex agricultural conditions such as afternoon light and haze. Furthermore, the SPPF-IS module enables additional feature fusion beyond the existing feature pyramid structure. The integration of these three modules significantly improves the model’s performance in detecting common diseases in potatoes and tomatoes.

It is believed that the methodologies and technologies developed in this study can be extended beyond potatoes and tomatoes to include the detection of leaf diseases in various crops, such as strawberries, apples, and wheat. For example, in strawberries, early detection of fungal diseases like Botrytis cinerea could help mitigate crop loss and improve yield quality. Similarly, in apple orchards, detecting early signs of apple scab or powdery mildew using this approach could reduce the need for chemical treatments, promoting more sustainable farming practices. Additionally, in wheat, where diseases like rust can lead to significant yield reductions, the application of these technologies could assist in real-time monitoring and targeted disease management.

These examples suggest a broader applicability for the development of smart agriculture technologies, enabling precision farming across a variety of crops. By incorporating such methods, farmers can optimize crop health monitoring, reduce pesticide use, and ultimately improve food security. This approach holds practical significance for advancing agricultural technologies and fostering more sustainable farming practices worldwide.

## 5 Conclusion

This paper introduces SIS-YOLOv8, a robust and efficient domain generalization model designed to detect common homologous diseases in potatoes and tomatoes within complex agricultural climatic contexts. The development of this model began with the collection of images through web scraping techniques, targeting early blight, late blight, and healthy leaves. These images were supplemented with those from the PlantVillage dataset and were manually annotated to enhance the dataset’s diversity and complexity by introducing simulated noise effects such as rain and smog, and performing image transformations like rotation, flipping, and translation.

The enhanced model, based on the YOLOv8 backbone and named SIS-YOLOv8, incorporates a novel convolutional architecture, Fusion-InceptionConv, which draws from the InceptionV1 architecture and the original YOLOv8n Conv module. This design significantly improves the model’s capability to extract detailed features under complex agricultural conditions, capturing finer-grained features and textures of diseased leaves, especially in environments with climatic interferences such as rain and smog.

Additionally, the study enhanced the YOLOv8n C2f layer with a Style Randomization feature, termed C2f-SIS, to aid in domain generalization. This enhancement, together with the upgraded InceptionV1+ module and the incorporation of the SimAM attention mechanism, allows the C2f layer to achieve multiscale feature fusion, enhance meaningful features, and suppress irrelevant ones, boosting the model’s contextual understanding and robustness under challenging climates.

Further advancements include the SPPF-IS layer, which enables additional multi-scale feature fusion within the existing feature pyramid structure and enhances feature decoupling at the final integration stage, augmenting the model’s robustness and generalization capabilities. To optimize performance, a Dependency Graph pruning method was employed to reduce the model’s parameter count and computational load without compromising accuracy.

Despite its successes, the study recognizes limitations and suggests future directions. The research currently focuses on early and late blights affecting potatoes and tomatoes, with potential expansions to include more crops and diseases. The relatively small dataset used could be expanded to enhance the model’s generalization capabilities. Furthermore, integrating this model with drone or robotic technologies could allow real-time monitoring of crop growth conditions, providing rapid diagnostics and precise localization of disease conditions, thereby reducing reliance on chemical treatments and enhancing agricultural productivity.

Overall, this study contributes significantly to the advancement of precision agriculture by providing an effective solution for the automatic detection of crop diseases, paving the way for future enhancements and broader applications in agricultural production.

## Data Availability

The original contributions presented in the study are included in the article/supplementary material. Further inquiries can be directed to the corresponding authors.
